# Prenatal exposure to alcohol does not affect radial maze learning and hippocampal mossy fiber sizes in three inbred strains of mouse

**DOI:** 10.1186/1744-9081-1-5

**Published:** 2005-04-22

**Authors:** Frans Sluyter, Laure Jamot, Jean-Yves Bertholet, Wim E Crusio

**Affiliations:** 1 Social, Genetic and Developmental Psychiatry Research Centre Institute of Psychiatry Kings College London, UK; 2 Trophos SA Parc Scientifique de Luminy – Case 931 13288 Marseille Cedex 09 France; 3 Institut de Psychologie Centre Henri Piéron Université de Paris V 71 avenue Edouard Vaillant 92100 Boulogne-Billancourt France; 4 Laboratoire de Neurosciences Cognitives, CNRS UMR 5106 Avenue des Facultés 33405 Talence France

**Keywords:** Prenatal Alcohol Exposure, Radial Maze Learning, Hippocampus, Mossy Fibers, Inbred Mouse Strains

## Abstract

**Background:**

The aim of this study was to investigate the effects of prenatal alcohol exposure on radial-maze learning and hippocampal neuroanatomy, particularly the sizes of the intra- and infrapyramidal mossy fiber (IIPMF) terminal fields, in three inbred strains of mice (C57BL/6J, BALB/cJ, and DBA/2J).

**Results:**

Although we anticipated a modification of both learning and IIPMF sizes, no such effects were detected. Prenatal alcohol exposure did, however, interfere with reproduction in C57BL/6J animals and decrease body and brain weight (in interaction with the genotype) at adult age.

**Conclusion:**

Prenatal alcohol exposure influenced neither radial maze performance nor the sizes of the IIPMF terminal fields. We believe that future research should be pointed either at different targets when using mouse models for Fetal Alcohol Syndrome (e.g. more complicated behavioral paradigms, different hippocampal substructures, or other brain structures) or involve different animal models.

## Background

It has long been known that prenatal exposure to alcohol can have devastating effects. In 1968, Lemoine et al. published a paper, in French, that described "des anomalies dans les infants de parents alcooliques" [1]. Five years later Jones and Smith [2] followed with their now classic paper on Fetal Alcohol Syndrome (FAS), which is the name given to a group of physical and mental birth defects that are the direct result of a woman's drinking alcohol during pregnancy. These defects may include mental retardation, growth deficiencies, central nervous system dysfunction, craniofacial abnormalities, and behavioral maladjustments (for an enumeration, see, among others, [3], and ).

Since then literally thousands of studies have been done and substantial advances in the understanding of FAS have been made, not in the least because of animal models, among them the mouse [4,5]. These models strongly parallel the physiological responses to alcohol in human development and have led to valuable insights into the susceptibility to alcohol of specific parts of the developing embryo. One of the more vulnerable brain regions, for instance, is the hippocampus, which is believed to be a primary target of prenatal alcohol and therefore responsible for many neurobehavioral abnormalities [6]. Thus, Riley et al. [7] found the effects of prenatal alcohol exposure on behavior to be similar to those of hippocampal lesions, while hippocampal dysfunction as a consequence of prenatal alcohol exposure has also been assessed in spatial learning tasks such as the Morris water navigation task (see, among others, [8]). However, little attention has been paid to the genetic vulnerability of the developing embryo to prenatal alcohol exposure, particularly genetically determined differences in the sensitivity of the developing hippocampus. These genetically determined differences might, at least partly, explain one of the major questions in the etiology of FAS: why only a small percentage of alcoholic women give birth to children with fetal FAS, whereas other alcoholic women who drink the same amount do not.

The aim of this study was to determine whether different genotypes with varying hippocampal anatomy are oppositely affected by similar exposures to prenatal alcohol. To this end, pregnant females of three highly inbred mouse strains, C57BL/6J, BALB/cJ, and DBA/2J, which vary in hippocampal anatomy (e.g. [9,10]), were exposed to a 12% alcohol solution as their only source of liquid throughout gestation. Following this, the male offspring was tested for their capacity to master an eight-arm radial maze, a spatial navigation task well known to depend on an intact hippocampus. Subsequently, the animals were sacrificed and the sizes of the hippocampal intra- and infrapyramidal mossy fiber (IIPMF) terminal fields were determined. Necessary control groups (untreated and pair-fed) were included.

## Results

Table 1 shows the numbers of breeding pairs used (pairings), the number of pairings resulting in pregnancies, and the number of pregnancies leading to live births in the nine different (3 strains × 3 groups) groups. Although ethanol exposed C57BL/6J females became pregnant at similar rates as animals from other treatment groups and strains, these pregnancies resulted in significantly lower live births than in the pair-fed and control groups (χ^2 ^= 9.5, df = 2; p < 0.01).

**Table 1:** Numbers of breeding pairs used (pairings), resulting pregnancies, and live births in the different groups

Strain/Treatment	# Pairings	# Pregnancies	# Births
			
**C57BL/6J**			
Ethanol	16	13	1*
pair-fed	10	7	5
Control	7	7	4
			
**BALB/cJ**			
Ethanol	16	14	8
pair-fed	11	9	8
Control	6	6	5
			
**DBA/2J**			
ethanol	8	8	6
pair-fed	8	8	5
control	12	11	7

* p < 0.05 compared to the C57BL/6J pair-fed and control groups. See text for details.

**Figure 1:**
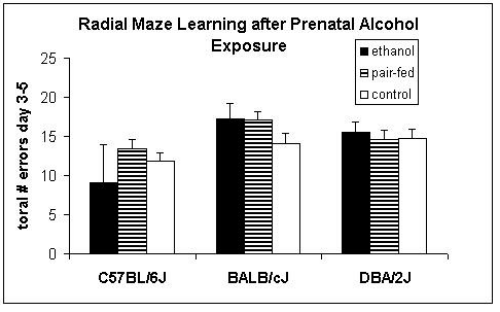
**Effects of prenatal alcohol exposure on total numbers of errors over the last three days of training in the radial-maze in male C57BL/6J, BALB/cJ, and DBA/2J mice**. *n *= 11–17 animals per group, except for C57BL/6J prenatally-exposed to ethanol (*n *= 2)

**Figure 2:**
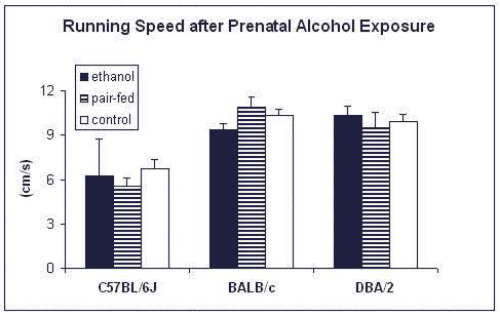
**Effects of prenatal alcohol exposure on running speeds (cm/s) in the radial-maze in male C57BL/6J, BALB/cJ, and DBA/2J mice**. *n *= 11–17 animals per group, except for C57BL/6J prenatally-exposed to ethanol (*n *= 2)

Figure 1 depicts the number of total errors (summed from day 3 up to day 5). Only the strain origin affected this variable (*F*_2,96 _= 5.0; p < 0.01). C57BL/6J made fewer errors than BALB/c (*z *= 3.2; *p *< 0.01) and DBA/2 (*z *= 2.5; *p *< 0.05). Treatment had no effect, neither alone, nor in interaction with strain. Figure 2 shows the running speeds in the radial maze. Similar to the number of errors, only the strain origin affected this variable (*F*_2,96 _= 13.9; *p *< 0.001) with again C57BL/6 males differing from the other two strains (vs. BALB/c: *z *= 5.0; *p *< 0.001; vs. DBA/2: *z *= 4.8; *p *< 0.001).

**Figure 3:**
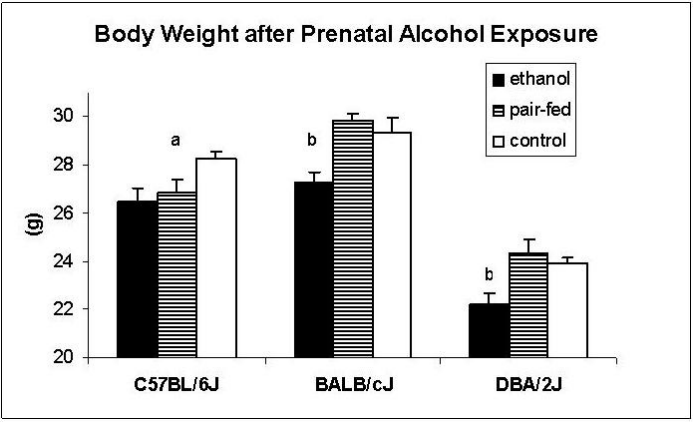
**Effects of prenatal alcohol exposure on body weight (g) in adult male C57BL/6J, BALB/cJ, and DBA/2J mice**. a: significantly different from controls (P < 0.05); b: Significantly different from controls (P < 0.01) and pair-feds (P < 0.001). *n *= 11–17 animals per group, except for C57BL/6J prenatally-exposed to ethanol (*n *= 2)

**Figure 4:**
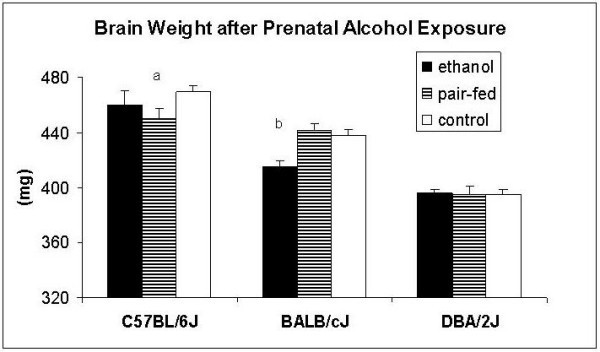
**Effects of prenatal alcohol exposure on brain weight (mg) in adult male C57BL/6J, BALB/cJ, and DBA/2J mice**. a: significantly different from controls (P < 0.01); b: Significantly different from controls (P < 0.01) and pair-feds (P < 0.001). *n *= 11–17 animals per group, except for C57BL/6J prenatally-exposed to ethanol (*n *= 2)

Body and brain weights are presented in Figures 3 and 4, respectively. The origin of the strain influenced both variables (body weight: *F*_2,96 _= 104.3; *p *< 0.001; brain weight: *F*_2,91 _= 86.1; *p *< 0.001). BALB/c weighed more than C57BL/6 (*z *= 3.1; *p *< 0.01) and DBA/2 (*z *= 14.2; *p *< 0.001). The latter strain, in turn, weighed less than C57BL/6 (*z *= 7.2; *p *< 0.001). C57BL/6 males had higher brain weights than BALB/c (*z *= 5.0; *p *< 0.001) and DBA/2 (z = 11.7; p < 0.001). In addition, BALB/c had higher brain weights than DBA/2 (z = 9.1; *p *< 0.001). Prenatal exposure to alcohol affected both variables. Body weights were affected independently of the origin of the genotype (*F*_2,96 _= 6.6; *p *< 0.01) with mice exposed to ethanol prenatally weighing less than control (*z *= 3.5; *p *< 0.01) and pair-fed (*z *= 3.2; *p *< 0.001) animals. The effect on brain weight depended on the background of the strain (strain × treatment: *F*_4,96 _= 3.8; *p *< 0.01). This interaction effect was mainly caused by the BALB/c strain where prenatal alcohol decreased brain weight (vs. pair-fed: *z *= 3.6; *p *< 0.001; vs. control: *z *= 3.1; *p *< 0.01), whereas no significant effects were seen in the other two strains.

**Figure 5:**
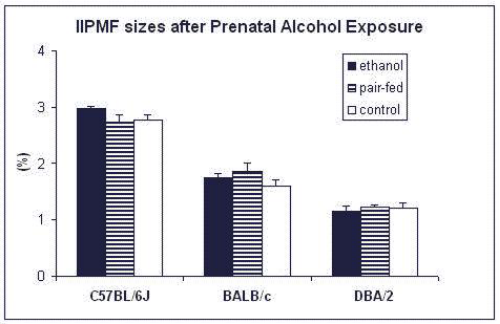
**Effects of prenatal alcohol exposure on the sizes of the IIPMF terminal fields (percentage of regio inferior) in male C57BL/6J, BALB/cJ, and DBA/2J mice**. For all groups n = 5, except for C57BL/6J prenatally-exposed to ethanol (*n *= 2).

The results of the hippocampal data are presented in Table 2 and Figure 5. Strain effects were observed for the following variables: regio inferior (*F*_2,73 _= 8.0; *p *< 0.001), stratum lacunosum-moleculare (*F*_2,73 _= 18.9; *p *< 0.001), stratum radiatum (*F*_2,73 _= 5.6; *p *< 0.01), suprapyramidal MF (*F*_2,73 _= 6.0; *p *< 0.01) and IIPMF (*F*_2,73 _= 132.0; *p *< 0.001). DBA/2 showed a smaller regio inferior than C57BL/6 (*z *= 3.7; *p *< 0.001) and BALB/c (*z *= 2.9; *p *< 0.001). C57BL/6 showed a larger stratum lacunosum-moleculare than DBA/2 (*z *= 6.1; *p *< 0.001) and BALB/c (*z *= 4.3; *p *< 0.001) while BALB/c males had a larger stratum lacunosum-moleculare than DBA/2 males (*z *= 2.4; *p *< 0.05). C57BL/6 exhibited smaller stratum radiatum and suprapyramidal MF than DBA/2 (*z *= 3.3; *p *< 0.01 and *z *= 3.4; *p *= 0.001) and BALB/c (*z *= 2.4; *p *< 0.05 and *z *= 2.8; *p *< 0.01). C57BL/6 showed larger IIPMF sizes than DBA/2 (*z *= 16.2; *p *< 0.001) and BALB/c (*z *= 10.8; *p *< 0.001). BALB/c had larger IIPMF sizes than DBA/2 (*z *= 7.0; *p *< 0.001).

**Table 2:** Sizes of hippocampal fields in the nine different groups Regio inferior (hilus + CA3) in 10^3 ^μm^2^, other hippocampal fields as percentage of regio inferior. Values represent means ± SEM. For statistical details, see text.

Strain/Treatment	Regio inferior	Stratum oriens	Stratum pyramidale	Stratum radiatum	Stratum lacunosum-moleculare	Hilus	Suprapyramidal Mossy Fibers
							
**C57BL/6J**							
Ethanol	753.9 ± 0.4	35.0 ± 0.05	15.2 ± 0.6	24.6 ± 0.1	8.1 ± 0.4	8.3 ± 0.9	8.7 ± 0.1
Pair-fed	775.4 ± 24.3	33.6 ± 0.8	14.7 ± 0.4	26.1 ± 0.4	7.9 ± 0.2	9.4 ± 0.2	8.5 ± 0.2
Control	747.8 ± 30.6	33.6 ± 0.5	14.8 ± 0.2	25.6 ± 0.6	8.3 ± 0.4	9.7 ± 0.3	8.3 ± 0.2
							
**BALB/cJ**							
Ethanol	726.9 ± 18.5	34.5 ± 0.6	14.7 ± 0.2	26.6 ± 0.2	6.5 ± 0.2	8.4 v 0.3	9.3 ± 0.4
Pair-fed	742.9 ± 27.7	33.6 ± 0.9	15.2 ± 0.3	26.1 ± 0.5	7.2 ± 0.3	7.9 ± 0.3	9.6 ± 0.4
Control	690.3 ± 23.9	34.3 ± 0.9	14.3 v 0.3	26.6 ± 0.2	6.8 ± 0.2	8.8 ± 0.3	9.3 v 0.3
							
**DBA/2J**							
Ethanol	638.0 ± 25.7	34.1 ± 0.5	15.0 ± 0.3	26.3 ± 0.3	6.6 ± 0.3	8.2 ± 0.3	9.7 ± 0.3
Pair-fed	649.9 ± 21.1	32.7 ± 0.6	15.3 ± 0.5	27.0 ± 0.3	6.0 ± 0.2	8.9 ± 0.4	10.0 ± 0.4
Control	699.6 ± 26.0	33.5 ± 0.5	15.2 ± 0.3	27.1 ± 0.5	6.4 ± 0.3	8.5 ± 0.2	9.4 ± 0.3

Prenatal alcohol exposure did not affect any of the hippocampal variables, neither as main factor, nor in interaction with the background.

## Discussion

These data demonstrate that, in three inbred strains of mice (C57BL/6, BALB/c, and DBA/2), prenatal exposure to alcohol does neither affect spatial memory nor hippocampal neuroanatomy. Prenatal alcohol exposure did influence body and brain weight in some strains and dramatically reduced live birth rates in C57BL/6 animals.

A first caveat is, of course, the observation that the low number (2) of C57BL/6 pups prenatally exposed to ethanol precludes any strong conclusions for that strain. We still decided to include these animals in the present report because simple visual inspection of the data shows that even for these two lone survivors there is not even a trend towards any differences in behavior or hippocampal morphology, just as is the case for the other two strains. Of course, sample sizes are much more adequate for strains BALB/c and DBA/2, so that the conclusions based on those strains are much stronger.

In short, our results on both behavior and neuroanatomy appear not to be in line with those, for instance, summarized in Berman and Hannigan [11], who concluded that prenatal alcohol exposure consistently produced significant deficits in spatial learning and/or memory. A closer look, however, revealed that most animal studies on the effects of prenatal alcohol exposure have been performed in rats and not mice. For instance, a survey of the recent literature, using PubMed, revealed only few studies that used mice as experimental models for prenatal alcohol exposure, and, to our knowledge, no studies involved multiple (>2) inbred mouse strains in the analysis of brain-behavior relations with regard to prenatal alcohol exposure. We could only find one study [12] that included two inbred strains (C57BL/10 and DBA/1), which reacted differently to early alcohol exposure. For instance, open field activity was decreased in C57BL/10, but not in DBA/1 mice, whereas aggression was more affected in DBA/1s. Most mouse studies, however, investigated the effects of prenatal alcohol exposure on various neurobehavioral aspects in only one strain, namely C57BL/6 mice. Thus, Opitz et al. [13] intubated pregnant C57BL/6 females with alcohol from gestational day 14–18 and investigated whether this affected radial maze performance in their offspring. As was the case in the present study, no effect of prenatal alcohol exposure on radial maze performance was found and it might, therefore, be concluded that radial maze performance is not affected by prenatal alcohol exposure in mice. One should keep in mind, though, that there might be other reasons why such an effect is not observed. One possibility is that blood alcohol levels were low in this study, an argument also raised by Opitz et al. in their discussion of their data [13]. Since we decided not to disturb pregnancies by taking blood samples (the rationale being that prenatal stress would then be a confounding factor), we were not able to determine blood ethanol concentrations (BAC). Hence, 'our' BACs might have been too low to have an effect. However, brain weights as well as body weights were affected in these experiments by the exposure to prenatal alcohol, a finding reminiscent of Wainwright et al. [14]. In addition, live births were dramatically reduced in C57BL/6, but the two male pups that we did obtain were phenotypically completely normal. It should furthermore be noted that the alcohol concentration used in the present study was almost three times as high as those used by previous authors [15,16], who reported significant effects on BACs (35-100mg/dl) in their female mice. A more recent study in which ethanol concentrations were slowly increased to 10% over pregnancy showed even higher BACs in the mother (varying from 50 to 150 mg/dl) [17]. Taken together these findings make it very unlikely that not sufficient ethanol reached the developing embryos to have significant effects.

Another explanation for the lack of an effect on learning might be that this type of radial maze is not sufficiently demanding and that other spatial learning tests would be more appropriate to detect prenatal alcohol effects. However, Wainwright et al. [14] did not observe any apparent effects of prenatal alcohol exposure on any measures of performance in a water navigation task in an F2 cross between C57BL/6 and DBA/2 males. Hence, other types of learning tests, less spatial ones such as the puzzle box [18], might perhaps reveal prenatal alcohol effects. In this respect it is interesting to note that in adult C57BL/6 mice prenatal alcohol exposure weakens the efficacy of reinforcers [15], impairs the development of conditioned taste aversion [13], and enhances the sensitivity to amphetamine [16]. We would also like to point out that the radial-maze task used here is quite sensitive and has been used successfully in the past to show learning defects that were not, or only barely, detectable in a water navigation task [19]. An alternative explanation for the absence of effects could be species-specific characteristics with mice being less vulnerable to alcohol per se than rats. It should be noted, however, that other treatments affecting hippocampal mossy fiber projections, such as early postnatal hyperthyroidism, have similar neuroanatomical (and behavioral) effects in rats and mice [20,21]. A final possibility is that effects depend on the age of testing. For instance, there might be a specific time window, in which the effects become visible. Either the effects might be transient or they might only appear at a later age (see [11] for a discussion).

Another striking result of this study is the low delivery rate of C57BL/6J females. Out of 13 pregnancies only one female gave birth to a viable litter. The other twelve pregnancies resulted in still-born pups, or the mother died while giving birth, or the pups were eaten by their mothers immediately after birth. Whether this finding reflects a higher sensitivity in developing C57BL/6J embryos to alcohol or higher blood alcohol concentrations in the mother or a combination of both cannot be inferred from these experiments. Although the only litter born might not be a representative sample, males from this litter appeared to perform equally well in the radial maze as their pair-fed and control counterparts; neither were the sizes of the IIPMF terminal fields affected.

## Conclusion

Summarizing, in this experimental design, which used three distinct inbred strains of mice, prenatal alcohol exposure influenced neither radial maze performance nor the sizes of the IIPMF terminal fields. We believe that future research should be pointed either at different targets when using mouse models for FAS (e.g. more complicated behavioral paradigms, different hippocampal substructures, or other brain structures) or involve different animal models, including zebrafish [22] or guinea-pig [23].

## Methods

### Animals

Subjects were male mice from the inbred strains C57BL/6J, BALB/cJ, and DBA/2J that are known to differ in their sensitivity to ethanol (e.g. [24]). The mice were kept under controlled laboratory conditions: temperature 24 ± 2°C; 12:12 light-dark schedule with lights on at 8:00 AM; room; food (IU UAR) and tap water ad libitum; dust-free sawdust bedding. Animals were weaned at 29 ± 1 days and each male was housed with a female (preferably a littermate) in a Plexiglas cage (42 cm × 27 cm × 17 cm). The experiments were performed at the University René Descartes (Paris V; CNRS URA 1294, Génétique, Neurogénétique et Comportement). All experimental animals were born and raised in the Paris animal facilities, which were SPF and approved by the French Ministry of Agriculture.

### Prenatal Alcohol Exposure

Eight days before mating, female mice of all three stains were habituated to alcohol solutions as their only source of liquid (4% ethanol the first 4 days, 8% ethanol the last 4 days). Females were then mated with experienced males from the same strain and, throughout gestation, exposed to a 12% ethanol solution as their only source of liquid. Two control groups were included: first, a pair-fed group which consumed an isocaloric solution of dextrin (Amisol) and a same amount of food as consumed by the alcohol-exposed dams and, second, a control group which received food and tap water ad libitum. Although food and liquid consumption were not specifically measured, no obvious differences in consumption were noticeable. It has been shown that voluntary drinking paradigms lead to significantly elevated blood alcohol levels in pregnant females [17]. Pregnancy was confirmed by visual inspection of females. After parturition offspring remained with their biological mother and tap water replaced the alcohol solution.

### Radial Maze Training

At the age of 3–4 months animals were tested in the radial maze. The 8-arm radial maze used in these experiments was similar to the original one used by Schwegler and Crusio (e.g. refs [25,26]). The central part of the radial-maze measured 20 cm in diameter. Its arms (25 cm long, 6 cm high, 6 cm wide) were closed and made of transparent Plexiglas. At the end of each arm was a perforated partition behind which fresh food pellets were deposited. In this way, the animals could not smell the presence or absence of a reward. All arms were reinforced by placing a small food pellet behind a low barrier preventing the animal from seeing whether a specific arm was still baited or not. The maze was always oriented in space in the same way. Several extra-maze cues were provided close to the arms. A confinement procedure was used utilizing transparent guillotine doors at the entrance of each arm. The doors were lowered and kept closed for 5 seconds after animals returned to the central box. The radial maze was placed directly on the floor to avoid possible elevation-induced anxiety.

Twenty-four hours prior to the experiment animals were moved to the test room. Animals were habituated for 1 day and subsequently trained for 5 days. The habituation consisted of a 15-min exploration trial with free access to all arms but without a food reward. Immediately afterwards companion females were removed from the home cages and all experimental animals, now single housed, were deprived of food. During the training sessions animals were weighed daily and kept at 80–90% of their original body weight. In between sessions the maze was cleaned with a dry cloth. On the first two days, trials were terminated after 15 minutes or after the animal had eaten all rewards, whichever came first. Thereafter, no time limit was imposed and trials were terminated when animals had found all 8 food rewards. The situation of animals not eating all rewards occurred frequently on the first two days, but never on days 3 to 5. For this reason, data from days 1 and 2 were not included in the analyses. Previous experiments have shown that significant learning occurs very rapidly and that strain or mutational effects can reliably be shown with this method [27]

Two variables were sampled, one representing learning performance and the other running speed. Learning was measured by the number of errors while activity was depicted as the mean distance traveled (cm) per second. An error was counted if an animal entered an arm previously visited or did not eat the reward. Average running speed was estimated by dividing the distance traveled by the amount of time needed to complete a trial.

### Histology and Morphometry

Histological treatments were performed as previously described by Schwegler and Lipp [28] see also [29]. Briefly, mice were deeply anesthetized and perfused intracardially with sodium sulfide and glutaraldehyde. This method allows a good fixation and preparation of the tissue for Timm's stain. Brains were removed, weighed, and post-fixed 24 hours in 3% glutaraldehyde with 20% sucrose and subsequently cut horizontally in 40 μm cryostat sections after which Timm's silver sulfide staining was applied.

**Figure 6:**
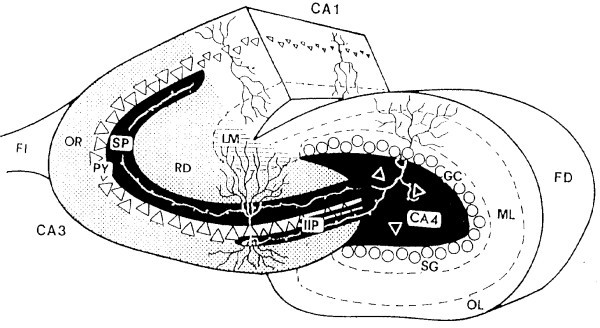
**Diagram of a Timm-stained cross-section of the hippocampus**. The hippocampal subregion CA3-CA4 (the area of morphometry) is indicated in black, stippled, and hatched areas. Black areas: suprapyramidal (SP), intra- and infrapyramidal (IIP) and hilar (CA4) mossy fiber terminal fields originating from the dentate gyrus. Stippled area: strata oriens (OR) and radiatum (RD). Hatched area: stratum lacunosum-moleculare (LM). CA1, subregion of the hippocampus without mossy fibers; FI, fimbria hippocampi; FD, fascia dentata; OL and ML, outer and middle molecular layers of the fascia dentata; SG, supragranular layer; GC, granular cells.

Methods used for visualization and measurements of the hippocampal terminal fields were similar to those described previously (e.g. [30]). Sampling started at the midseptotemporal level, directly below the most ventral extension of the septal pole of the fascia dentata. Taking every second section, 5 defined horizontal sections per animal were pseudo-randomly sampled, alternating between the left and right hippocampus. Areas of the strata oriens, pyramidale, radiatum, lacunosum-moleculare, and the mossy fiber terminal fields (hilus, suprapyramidal MF, and intra- and infrapyramidal MF; see Figure 6) were measured on an image analyzing system (Samba, Alcatel) and were expressed as a percentage of the whole regio inferior (hilus + CA3) to correct for possible slight variations in cutting plane or tissue shrinkage. This standardized method has been shown to yield reliable and replicable results [28].

### Statistical analysis

Using χ^2^-tests the number of breeding pairs used (pairings), pregnancies, and births were compared for each strain. Pregnancies were compared relative to the number of pairings, and births relative to the number of pregnancies. The radial maze data of days 3 to 5 were analyzed using two-way repeated measures ANOVAs with strain and treatment as main factors. Both between subjects factors consisted of three levels (strain: C57BL/6J, BALB/cJ, and DBA/2J; treatment: alcohol exposed, pair-fed, and control). The hippocampal data were analyzed by means of two-way ANOVAs, both factors being identical to the above-mentioned analysis. When necessary, pair-wise comparisons were made using least square means. All ANOVAs were performed using the SAS GLM procedure.

## Authors' Contributions

FS participated in the interpretation of the data and drafted the manuscript, LJ carried out the radial maze tests and performed the histology, JYB participated in the design of the study and carried out the morphometrical analyses, and WEC conceived of the study, participated in its design, carried out the statistical analyses, and participated in the interpretation of data and drafting of the manuscript. All authors read and approved the final manuscript.
